# Retraction: Nesfatin-1 regulates the phenotype transition of cavernous smooth muscle cells by activating PI3K/AKT/mTOR signaling pathway to improve diabetic erectile dysfunction

**DOI:** 10.1371/journal.pone.0314890

**Published:** 2024-12-02

**Authors:** The PLOS ONE Editors

The *PLOS ONE* Editors retract this article due to concerns about duplicate publication.

Following the publication of this article [1] the author requested retraction for issues involving authorship and scientific concerns. During the editorial investigation into this matter, PLOS noted that the article was previously published in [2]. In light of the duplicate publication concerns, PLOS did not investigate the merit of the authorship and scientific concerns further.

KC, BH, JF, ZH, SF, SR, HT, AMAMM, XW, YT, HX, RH, and GL agreed with the retraction. KC, JF, ZH, XW, and GL apologize for the issues with the article. JF stands by the article’s findings. XL either did not respond directly or could not be reached.
